# Impact of sociodemographic factors on outcomes in patients with peritoneal malignancies following cytoreduction and chemoperfusion

**DOI:** 10.1002/jso.26843

**Published:** 2022-03-06

**Authors:** Adriana Cantos, Emanuel Eguia, Xuanji Wang, Gerard Abood, Lawrence M. Knab

**Affiliations:** ^1^ Department of Surgery Loyola University Chicago Stritch School of Medicine Maywood Illinois USA; ^2^ Department of Surgery Loyola University Medical Center Maywood Illinois USA

**Keywords:** cytoreductive surgery, heated intraperitoneal chemotherapy, social disparities, socioeconomic status

## Abstract

**Background and Objectives:**

Sociodemographic factors have been shown to impact surgical outcomes. However, the effects of these factors on patients undergoing cytoreductive surgery (CRS) and heated intraperitoneal chemotherapy (HIPEC) are not well known. This study aims to evaluate the impact of sociodemographic factors on patients undergoing CRS/HIPEC.

**Methods:**

Adult patients at a tertiary center who underwent CRS/HIPEC were evaluated. Perioperative variables were collected and analyzed. A national database was also used to evaluate patients undergoing CRS/HIPEC.

**Results:**

There were 90 patients who underwent CRS/HIPEC (32% non‐White). There was no statistically significant difference in postoperative complications, length of stay, or discharge disposition based upon race (white vs. non‐White patients), socioeconomic status (SES), or insurance type. Nationally, we found that Black and Hispanic patients were less likely to undergo CRS/HIPEC than Non‐Hispanic white patients (Black: odds ratio [OR]: 0.60, [confidence interval {CI}: 0.39–0.94]; Hispanic: OR: 0.52, [CI: 0.28–0.98]). However, there were no significant differences in postoperative complications based upon race/ethnicity.

**Conclusion:**

Sociodemographic factors including race, SES, and insurance status did not impact postoperative outcomes in patients undergoing CRS/HIPEC at our single institution. On a national level, Black and Hispanic patients underwent CRS/HIPEC at lower rates compared to white patients.

## 1 INTRODUCTION

Sociodemographic factors such as race/ethnicity, language proficiency, socioeconomic status (SES), education level, and sex play a crucial role in health outcomes. Social determinants of health have been shown to negatively impact surgical outcomes, including increased postoperative complications, morbidity, mortality, and readmissions. Access to appropriate care is an additional barrier faced by disadvantaged populations. Ultimately, these patients may not be offered or receive the surgical standard of care due to a variety of factors.

Multiple studies have shown that Black patients have worse outcomes after surgery compared to white patients.^1^, ^2^, ^3^, ^4^, ^5^, ^6^ Minority status, geographic location, and insurance type have also been associated with worse postoperative outcomes in a variety of gastrointestinal malignancies.^7^, ^8^, ^9^, ^10^, ^11^ A Surveillance, Epidemiology, and End Results (SEER) database study involving patients with pancreatic adenocarcinoma showed that while Black and white patients presented with a similar stage of disease and were recommended surgery at a similar rate, Black patients underwent fewer resections and had worse overall survival.^12^ Other studies have demonstrated that for early stage pancreatic adenocarcinomas, Black patients are offered resections at lower rates compared to white patients.^7^ Similar disparities persist in the treatment of ovarian cancer.^13^, ^14^, ^15^, ^16^

While multiple studies have examined the impact of social determinants of health on gastrointestinal malignancies, few studies have examined the effect specifically on patients with peritoneal malignancies undergoing cytoreductive surgery (CRS) and heated intraperitoneal chemotherapy (HIPEC).^17^ This patient population is uniquely vulnerable due to the high morbidity and mortality of the required operations, in addition to the complex pre‐ and postoperative care that is often needed to successfully care for these patients. They often have severe protein malnutrition perioperatively, and can have complex home‐care needs including ostomies, drains, nutritional supplements, and wound care.

The goal of this study was to examine a single‐institution experience of patients with peritoneal malignancies undergoing cytoreduction and chemoperfusion, and determine if social disparities impacted postoperative outcomes. Additionally, a national database was examined to determine if social disparities impacted access to this complex surgical procedure.

## 2 METHODS AND METHODS

### 2.1 Study design

As approved by the Loyola University Medical Center Institutional Review Board (IRB), a retrospective chart review was performed for all patients with peritoneal malignancies who underwent CRS/HIPEC from April 2013 to February 2017 at our large, tertiary care hospital. Demographic information collected included race, insurance type (Medicaid, Medicare, private, uninsured), zip code, primary language, use of interpreter services, body mass index (BMI), and comorbidities.

Using the United States Census Bureau data from the American Community Survey (ACS) 5‐year estimates (2013–2017), median household income levels for each patient were derived from their zip code. As a measure of a patient's SES, Area Deprivation Index (ADI) values were also derived from each patient's zip code. The ADI is a novel tool based on the United States Census and ACS Data. This is a standardized, multidimensional evaluation of an area's socioeconomic condition based on 17 variables, including housing, income, and education.^18^ High ADI values indicate high levels of socioeconomic deprivation, where an ADI of 100 indicates the most disadvantaged. For this study, ADI values > 25 were considered socioeconomically disadvantaged.

Analyses were also performed using the US Agency for Healthcare Research and Quality Healthcare Cost and Utilization Project's (HCUP) National Inpatient Sample (NIS). HCUP NIS is the largest all‐payer (Medicare, Medicaid, Private, and Uninsured) inpatient care database in the United States, is designed to be representative of all community hospitals, and is intended to be used for national estimates. Community hospitals are defined as short‐term, nonfederal, and nonrehabilitation hospitals. The NIS is drawn from a sampling frame that contains hospitals that have more than 95% of all discharges from statewide data organizations that contribute to HCUP. The makeup of NIS is a weighted sample of the State Inpatient Databases in a single‐cluster design stratified on geographic area, urban/rural, ownership, teaching status, and bed size. The NIS is also standardized across years to facilitate trend analyses although the states contributing to the NIS vary from year to year. The odds of undergoing HIPEC are representative of the NIS population. Minority patient populations were normalized to the US population to calculate odds ratios. The NIS does not report the number of patients that were surgical candidates and not offered surgery, or those offered surgery but refused.

The study population is comprised of adult hospitalizations (≥18 years) in 2014. The identification of the cohort of hospitalizations was identified by a primary International Classification of Diseases, Ninth and Tenth Revision, Clinical Modification (ICD‐9) for HIPEC at the time of discharge as was done in previous studies.^19^ Patients with an ICD‐9 code 99.85 or both codes 54.97 and 99.25 were considered possible intraperitoneal chemotherapy cases. Code 99.85 was considered HIPEC, while the combination of codes 54.97 and 99.25 was considered non‐HIPEC. Potential cases were only included if they underwent an intra‐abdominal operation using the HCUP Clinical Classification Software for Services and Procedures codes (codes 66, 72–76, 78–80, 83, 87, 89–90, 94, 96, 99, 104, 112, 114, 119, 120, 123–125, and 132).^19^

### 2.2 Outcomes

Primary outcomes of interest focused on the length of stay, intensive care unit (ICU) days, disposition, and readmissions. Secondary outcomes of interest included postoperative complications such as delayed gastric emptying, infections, organ insufficiency, fistulas, and postoperative leak. We also evaluated patients who were underinsured and insured (only private insurance) and compared patient demographics and postoperative outcomes. The underinsured group included those patients with Medicare, Medicaid, and uninsured.

### 2.3 Statistical analysis

Our unadjusted comparison of two or more proportions were performed using a chi‐squared test and continuous variables were compared using a *t* test or Wilcoxon rank sum test as appropriate. Statistical significance was established at *α* = 0.05. In the HIPEC cohort, baseline characteristics of hospitalizations were presented using survey‐adjusted counts and means to provide national estimates with standard errors. Logistic regression analysis was also used to compare the odds of undergoing HIPEC by race/ethnicity. All regression analyses controlled for the following variables: age, sex, and Elixhauser mortality score. All analyses were performed using STATA 14 software. This study was evaluated and approved by the Loyola University Chicago IRB (#212524).

## 3 RESULTS

A total of 90 patients underwent CRS/HIPEC for all peritoneal malignancies during the study period. Patient demographics are presented in Table 1. Of the 90 patients, 68% were classified as Non‐Hispanic white, while 32% were classified as non‐White, which included Non‐Hispanic Black, Hispanic, Non‐Hispanic Asian, and Non‐Hispanic other. In the group of Non‐Hispanic white patients, 98% spoke English as their primary language, compared to 62% of patients in the non‐White group. An interpreter was used in 28% of patient encounters in the minority group. There were no significant differences between the two groups in age (*p* = 0.14), sex (*p* = 0.64), BMI (*p* = 1.00), ASA class (*p* = 0.71) or insurance type (*p* = 0.80). Median household income was significantly higher in the Non‐Hispanic white group (mean ± *SD* = 77 939 ± 3165) versus the non‐White group (mean ± *SD* = 64 060 ± 4351) (*p* = 0.01).

**Table 1:** Patient characteristics

	Non‐Hispanic White		Non‐White		*p*
No. patients, *n* (%)	61	68%	29	32%	
Age years, mean (*SD*)	59	1.68	54	3.06	0.14
Male, *n* (%)	22	36%	9	31%	0.64
Race/ethnicity, *n* (%)					
Non‐Hispanic White	61	100%	0	0%	
Non‐Hispanic Black			6	21%	
Hispanic			6	21%	
Non‐Hispanic Asian			7	24%	
Non‐Hispanic Other			9	31%	
Insurance, *n* (%)					
Private	32	52%	15	52%	0.80
Medicare	24	39%	10	34%	
Medicaid	3	5%	3	10%	
Uninsured	2	3%	1	3%	
Income, mean (*SD*)	$77 939	$3165	$64 060	$4351	0.01
BMI, mean (*SD*)	27	0.85	27	1.05	1.00
Language, *n* (%)					
English	60	98%	18	62%	0.00
Spanish	0	0%	5	17%	
Other	1	2%	6	21%	
Interpreter, *n* (%)	1	2%	8	28%	0.00
ASA, *n* (%)					
2	3	5%	2	7%	0.71
3	48	79%	24	83%	
4	10	16%	3	10%	
Low SES, *n* (%)	36	59%	25	86%	0.36
Comorbidities, *n* (%)					
CVD	2	3%	1	3%	0.97
COPD	6	10%	1	3%	0.29
CHF	4	7%	0	0%	0.16
DM	2	3%	3	10%	0.17
MI	1	2%	1	3%	0.59
PVD	8	13%	2	7%	0.38

Abbreviations: BMI, body mass index; CHF, congestive heart failure; CVD, cerebral vascular disease; DM, diabetes mellitus; HIV, human immunodeficiency virus; MI, myocardial infarction; PVD, peripheral vascular disease; SES, socioeconomic status.

Postoperative complications were examined based upon race, ADI scores, and insurance status. There were no significant differences in postoperative complications between Non‐Hispanic white and non‐White groups (Table 2A), low ADI patients compared to the high ADI patients (Table 2B), or insured versus underinsured (Table 2C). Additionally, the mean ICU days, length of stay (LOS), 30‐day readmission rates, and disposition were similar between racial, ADI, and insurance groups (Table 3A–C). Furthermore, there were no statistically significant differences in postoperative complications when language proficiency was evaluated. When complications were combined into one variable to increase the number, there was again no difference when stratified by race/ethnicity, ADI, or insurance.

**Table 2:** A. Postoperative complications by race/ethnicity

	Non‐Hispanic White		Non‐White		*p*
Total patients (*n*)	61		29		
Delayed gastric emptying	2	3%	0	0%	0.32
SSI	5	8%	1	3%	0.40
Pulmonary insufficiency	5	8%	6	21%	0.09
Ileus	12	20%	2	7%	0.12
Sepsis	13	21%	4	14%	0.39
Anastomotic leak	2	3%	2	7%	0.44
Thromboembolic event	1	2%	3	10%	0.33
Renal insufficiency	0	0%	1	3%	0.15
EC fistula	1	2%	0	0%	0.49
Biliary leak	0	0%	1	3%	0.15
Cardiac event	4	7%	4	14%	0.27
UTI	9	15%	6	21%	0.48

B. Postoperative complications by area deprivation index					
	Low (ADI ≤25)		High (ADI >26)		*p*
Total patients (*n*)	56		34		
Delayed gastric emptying	1	2%	1	3%	0.72
SSI	3	5%	3	9%	0.52
Pulmonary insufficiency	9	16%	2	6%	0.15
Ileus	7	13%	7	21%	0.31
Sepsis	9	16%	8	24%	0.38
Anastomotic leak	1	2%	3	9%	0.12
Thromboembolic event	3	5%	1	3%	0.63
Renal insufficiency	1	2%	0	0%	0.43
EC fistula	0	0%	1	3%	0.43
Biliary leak	0	0%	1	3%	0.20
Cardiac event	5	9%	3	9%	0.58
UTI	11	20%	4	12%	0.33

C. Postoperative complications by insurance status					
	Underinsured		Insured		*p*
Number of patients (*n*)	43		47		
Delayed gastric emptying	0	0%	2	2%	0.17
SSI	3	7%	3	6%	0.91
Pulmonary insufficiency	6	14%	5	11%	0.63
Ileus	8	19%	6	13%	0.45
Sepsis	6	14%	11	23%	0.25
Anastomotic leak	1	2%	3	6%	0.35
Thromboembolic event	3	7%	1	2%	0.30
Renal insufficiency	1	2%	0	0%	0.29
EC fistula	1	2%	0	0%	0.29
Biliary leak	0	0%	1	2%	0.34
Cardiac event	4	9%	4	9%	0.79
UTI	9	21%	6	13%	0.30

*Note*: High ADI indicates a low socioeconomic status.

Abbreviations: ADI, area deprivation index; EC, enterocutaneous; SSI, surgical site infection; UTI, urinary tract infection.

**Table 3:** A. Postoperative outcomes by race/ethnicity

	Non‐Hispanic White		Non‐White		*p*
Number of patients (*n*)	61		29		
ICU days, mean (*SD*)	5	5	6	11	0.27
LOS, mean (*SD*)	13	10	15	14	0.39
Disposition, *n* (%)					
Home	38	62%	19	66%	0.35
Rehabilitation facility	10	16%	5	17%	
Home health	11	18%	2	7%	
Died	0	0%	1	3%	
Unknown	2	3%	2	7%	
30‐day readmission, *n* (%)	15	25%	7	24%	0.96

B. Postoperative outcomes by area deprivation index (ADI)					
	Low		High		*p*
Number of patients (*n*)	56		34		
ICU days, mean (*SD*)	5	0.98	6	1.16	0.36
LOS, mean (*SD*)	14	1.46	14	2.08	0.99
Disposition, *n* (%)					
Home	32	57%	25	74%	0.43
Rehabilitation facility	12	21%	3	9%	
Home health	8	14%	5	15%	
Died	1	2%	0	0%	
Unknown	3	5%	1	3%	
30‐day readmission, *n* (%)	11	20%	11	32%	0.17

C. Postoperative outcomes by insurance status					
	Underinsured		Insured		*p*
Number of patients (*n*)	43		47		
ICU days, mean (*SD*)	5	8	5	6	0.97
LOS, mean (*SD*)	13	10	14	12	0.71
Disposition, *n* (%)					
Home	25	58%	32	68%	0.14
Rehabilitation facility	11	26%	4	9%	
Home health	4	9%	9	19%	
Died	1	2%	0	0%	
Unknown	2	5%	2	4%	
30‐day readmission, *n* (%)	9	21%	13	28%	0.46

Abbreviations: ICU, intensive care unit; LOS, length of stay.

The NIS database was used to identify 900 patients nationally who underwent CRS/HIPEC in 2014 (Table 4). The mean age was 55 with a mean Elixhauser Comorbidity score of 4. Of the 900 patients, 72.8% were white 10% Black, 6.1% Hispanic, and 7.8% other. The most common insurance type was private at 66.1%, followed by Medicare at 17.8%. The majority of operations were carried out at urban academic medical centers (95.6%).

**Table 4:** 2014 National estimates of HIPEC patient characteristics

Total patients, *n*	900	
Age, *mean* (*SE*)	54.8	1.6
Female, *n* (%)	500	55.6%
Elixhauser, *mean* (*SE*)	4	0.14
Race/ethnicity, *n* (%)		
Non‐Hispanic White	655	72.8%
Non‐Hispanic Black	90	10.0%
Hispanic	55	6.1%
Non‐Hispanic Other	70	7.8%
Missing	30	3.3%
Insurance, *n* (%)		
Medicare	160	17.8%
Private	595	66.1%
Medicaid	95	10.6%
Self‐pay	10	1.1%
No charge	0	0.0%
Other	40	4.4%
Missing	0	0.0%
Hospital characteristics, *n* (%)		
Rural	0	0.0%
Urban	40	4.4%
Academic Urban	860	95.6%
LOS, *mean* (*SE*)	11	0.72
In‐hospital death, *n (%)*	15	1.7%

*Note*: Elixhauser risk score for mortality and length of stay (LOS)

Cost inflated to 2016 Sepsis criteria adapted from Angus et al.^20^ other includes other, bone/joint, and devices.

When postoperative complications including sepsis, deep vein thrombosis, myocardial infarction, and pulmonary embolism were examined, there were no significant differences between racial/ethnic groups. The mean LOS for patients undergoing HIPEC was 11 days and there was no difference between the racial/ethnic groups.

In our risk adjusted analysis, adjusted for sex, age and comorbidities we found there were lower odds of undergoing CRS/HIPEC nationally for Non‐Hispanic Black (odds ratio [OR]: [95% confidence interval {CI}] = 0.60 [0.39–0.94]) and Hispanic (OR [95% CI] = 0.52 [0.28–0.98]) patients when compared to Non‐Hispanic white patients.

## 4 DISCUSSION

Social demographics including race, SES, insurance type, language proficiency, and education have been shown to affect access to surgical care as well as impact postoperative outcomes. In this study examining social disparities in patients undergoing CRS/HIPEC, we found that there were no significant differences in postoperative complications, LOS, disposition, or 30‐day readmissions when compared by race, SES, or insurance type. Analysis of a national database also did not demonstrate a statistically significant difference in postoperative complications when racial groups were compared, although it did demonstrate a decreased odds ratio of Black and Hispanic patients undergoing CRS/HIPEC compared to white patients.

There is relatively little published about social disparities and CRS/HIPEC, although one of the largest to date is by Rieser et al.^17^ who studied the impact of SES on 226 patients who underwent CRS/HIPEC for colorectal peritoneal metastases. They found that high‐SES patients were more likely to be white, privately insured, and had fewer comorbidities. Additionally, the data suggested that low‐SES patients had worse postoperative outcomes after CRS/HIPEC, including longer LOS, more complications, and more readmissions. These results differ from our findings, which did not demonstrate a difference between races when SES status was compared. Furthermore, our cohort did not have worse postoperative outcomes based upon race or SES.

Similarly, several studies have examined the impact of insurance status on postoperative outcomes in patients undergoing CRS/HIPEC. Chokshi et al.^20^ suggested that insurance status was not correlated to overall survival. In contrast, Hanna et al found that insurance status in CRS/HIPEC patients did impact outcomes, including increased complications and lower overall survival in underinsured patients.^21^ Our study did not demonstrate a significant difference in postoperative outcomes based upon insurance status. This coincides with the fact that we found no differences based upon SES either. Social determinants of health are complex, and other factors including food security, housing stability, transportation, and social support at home are just a few pieces of a larger puzzle that impact patient outcomes. Future work will focus on these other patient factors in addition to race, SES, and insurance type to compile a more detailed picture of the patient's risk factors.

Several large national database studies have demonstrated worse postoperative outcomes in cancer patients of low SES undergoing surgery.^22^ A SEER database study examining rates of surgical resection in patients with pancreatic cancer demonstrated that Black and white patients were offered surgical resection at equal rates although Black patients underwent fewer resections compared to white patients. For those patients that did undergo surgery, survival rates were similar between the races. Studies examining other cancer types have similar conclusions. For example, Black patients have been found to undergo surgery for esophageal, lung, prostate, and hepatocellular cancers at lower rates compared to white patients.^9^, ^23^, ^24^, ^25^, ^26^

Similarly, our data demonstrates that Black and Hispanic patients have a lower odds ratio of undergoing CRS/HIPEC. It is unclear if patients are not offered surgery or if patients are refusing the surgical option. This has not been studied in the past and is difficult to track because patients with peritoneal malignancies are often treated at multiple institutions before undergoing CRS/HIPEC and many times an index operation (e.g., a colectomy for colon cancer) will be performed years before the CRS/HIPEC to treat peritoneal metastases. Our data highlight an important racial disparity although further investigation is needed to determine the factors contributing to this.

This study has several limitations. It is retrospective from a single institution, and while the number of patients is relatively high for this disease process, it may be underpowered to show differences in certain patient populations. For example, due to the relatively low number of patients with limited English proficiency, it is difficult to determine significant differences between groups of patients based upon language proficiency. The use of the ADI to approximate SES shares limitations with the Census data including undocumented immigrant populations. This does add some error to the SES approximations, although the ADI has been well‐validated at the neighborhood level in prior studies. Additionally, the national database is excellent for broad conclusions, although the patient details are limited. While we know that minority patients have a lower odds ratio of undergoing CRS/HIPEC, further institutional studies are needed to determine why this is happening.

## 5 CONCLUSIONS

In conclusion, we did not find that sociodemographic factors including race, SES, ADI, or insurance status impacted postoperative outcomes in patients undergoing CRS/HIPEC at our single institution. We did find that on a national level, Non‐Hispanic Black and Hispanic patients underwent CRS/HIPEC at lower rates compared to white patients. Further investigation is needed to determine what factors account for this disparity.

## SYNOPSIS

The impact of socioeconomic status in patients undergoing cytoreductive surgery (CRS) and heated intraperitoneal chemotherapy (HIPEC) is not well known. We found that socioeconomic status did not impact postoperative outcomes at a single institution. Nationally, non‐White patients underwent CRS/HIPEC at lower rates compared to white patients.

## Data Availability

The data that support the findings of this study are available from the corresponding author upon reasonable request.
